# Development and Characterization of a Novel Wheat–Tetraploid *Thinopyrum elongatum* 6E (6D) Disomic Substitution Line with Stripe Rust Resistance at the Adult Stage

**DOI:** 10.3390/plants12122311

**Published:** 2023-06-14

**Authors:** Biran Gong, Lei Zhao, Chunyan Zeng, Wei Zhu, Lili Xu, Dandan Wu, Yiran Cheng, Yi Wang, Jian Zeng, Xing Fan, Lina Sha, Haiqin Zhang, Guoyue Chen, Yonghong Zhou, Houyang Kang

**Affiliations:** 1 State Key Laboratory of Crop Gene Exploration and Utilization in Southwest China, Sichuan Agricultural University, Chengdu 611130, China; biranwheat@163.com (B.G.); zhaoleizby@163.com (L.Z.); zeng97977@163.com (C.Z.); zhuwei202209@163.com (W.Z.); 14646@sicau.edu.cn (D.W.); chengyiran@sicau.edu.cn (Y.C.); wangyi@sicau.edu.cn (Y.W.); fanxing9988@163.com (X.F.); guoyuech74@hotmail.com (G.C.); zhouyh@sicau.edu.cn (Y.Z.); 2 Triticeae Research Institute, Sichuan Agricultural University, Chengdu 611130, China; xulili_0627@126.com; 3 College of Resources, Sichuan Agricultural University, Chengdu 611130, China; zengjian@sicau.edu.cn; 4 College of Grassland Science and Technology, Sichuan Agricultural University, Chengdu 611130, China; rice_shazhi@163.com (L.S.); haiqinzhang@163.com (H.Z.)

**Keywords:** chromosomal substitution, molecular markers, stripe rust, tetraploid *Thinopyrum elongatum*

## Abstract

Stripe rust, which is caused by *Puccinia striiformis* f. sp. *tritici*, is one of the most devastating foliar diseases of common wheat worldwide. Breeding new wheat varieties with durable resistance is the most effective way of controlling the disease. Tetraploid *Thinopyrum elongatum* (2n = 4x = 28, EEEE) carries a variety of genes conferring resistance to multiple diseases, including stripe rust, Fusarium head blight, and powdery mildew, which makes it a valuable tertiary genetic resource for enhancing wheat cultivar improvement. Here, a novel wheat–tetraploid *Th. elongatum* 6E (6D) disomic substitution line (K17-1065-4) was characterized using genomic in situ hybridization and fluorescence in situ hybridization chromosome painting analyses. The evaluation of disease responses revealed that K17-1065-4 is highly resistant to stripe rust at the adult stage. By analyzing the whole-genome sequence of diploid *Th. elongatum*, we detected 3382 specific SSR sequences on chromosome 6E. Sixty SSR markers were developed, and thirty-three of them can accurately trace chromosome 6E of tetraploid *Th. elongatum,* which were linked to the disease resistance gene(s) in the wheat genetic background. The molecular marker analysis indicated that 10 markers may be used to distinguish *Th. elongatum* from other wheat-related species. Thus, K17-1065-4 carrying the stripe rust resistance gene(s) is a novel germplasm useful for breeding disease-resistant wheat cultivars. The molecular markers developed in this study may facilitate the mapping of the stripe rust resistance gene on chromosome 6E of tetraploid *Th. elongatum*.

## 1. Introduction

*Thinopyrum elongatum* (Host) D. R. Dewey is a wild relative of wheat and possesses many desirable traits useful for improving wheat, including resistance to various diseases (e.g., stripe rust, Fusarium head blight, and powdery mildew) and salt stresses and tolerance to drought [1,2,3]. The three ploidy levels of this species are as follows: diploid (2n = 2x = 14, EE), tetraploid (2n = 4x = 28, EEEE), and decaploid (2n = 10x = 70, EEEEEEStStStSt) [4]. Research on *Th. elongatum* began in the former Soviet Union and was followed by studies involving hybridizations between *Th. elongatum* and wheat in various countries. These studies resulted in many wheat–*Th. elongatum* lines, including addition [5,6], substitution [7,8,9,10], translocation [11,12], and introgression lines [13,14], which successfully introduced disease resistance genes carried by *Th. elongatum* into common wheat, such as *Yr69*, *Pm51*, *Fhb7*, *Lr19*, *Lr24*, *Lr29*, *Sr24*, *Sr25*, *Sr26*, and *Sr43* [15]. Although most of these lines are the progeny of crosses between wheat and diploid *Th. elongatum* or decaploid *Th. ponticum*, there are a few reports describing crosses involving tetraploid *Th. elongatum* [16]. To date, some of the major materials produced for breeding transfer of resistance genes in tetraploid *Th. elongatum* are still partially diploid and additional lines [17,18]; none of these materials can be used directly in wheat breeding. Therefore, it is urgent to develop wheat–tetraploid *Th. elongatum* resistance lines without genetic drag and with more stablility, as well as to transfer the resistance genes carried by tetraploid *Th. elongatum* into common wheat.

Stripe rust caused by *Puccinia striiformis* f. sp. *tritici* is one of the major biotic factors limiting grain production in most cool and humid wheat-growing regions [11]. Over 80% of the wheat cultivated worldwide is affected by stripe rust, leading to annual yield losses of 5.47 million tons [19]. Compared with methods involving the application of pesticides, the breeding of disease-resistant varieties is considered to be a more cost-effective and environmentally friendly method for controlling stripe rust [20]. The genetic resistance to stripe rust in wheat has been classified as all-stage resistance (ASR) and adult plant resistance (APR) [21]. APR is usually controlled by multiple genes, each with a minor or partial effect, and most well-characterized APR genes are effective against multiple races (i.e., nonspecific) and confer relatively durable resistance [22]. To date, 28 of the 84 officially named stripe rust resistance genes are APR genes, of which three originated from the progenitors and wild relatives of wheat, including *T. dicoccoides*, *T. durum*, and *Secale cereale* [23,24,25]. In addition, many temporarily named Yr genes and quantitative trait loci (QTLs) mediating APR from the relatives of wheat have been identified, including *YrM1225* from *Aegilops ventricosa* [26,27,28]. Some of these resistance genes, such as *Yr36* from *T. dicoccoides*, which confers durable resistance to multiple *Pst* isolates, have been important for increasing wheat production [29,30]. However, the rapid diversification of *Pst* races has resulted in the emergence of new virulent *Pst* races that can overcome the effects of most of the stripe rust resistance genes [31]. More specifically, the partial or complete resistance provided by *Yr1*, *Yr2*, *Yr3*, *Yr4*, *Yr6*, *Yr7*, *Yr9*, *Yr10*, *Yr17*, *Yr22*, *Yr23*, *Yr26*, and *Yr27* has been lost in most regions of the globe [32,33]. Therefore, exploring new germplasm resources and exploiting their disease resistance genes are critical for genetically improving wheat.

In a previous study, Li et al. [34] developed 50 wheat–tetraploid *Th. elongatum* derivative lines by crossing the *T. durum*–tetraploid *Th. elongatum* partial amphidiploid line 8801 with Sichuan wheat cultivars. More than 70% of these lines were highly resistant to stripe rust, unlike the parental wheat cultivars. We subsequently further developed and characterized the wheat–tetraploid *Th. elongatum* 1E (1D), 3E (3D), and 4E (4D) disomic substitution lines, which revealed the successful transfer of salt tolerance genes and genes for ASR to stripe rust and powdery mildew from tetraploid *Th. elongatum* into common wheat [16,35,36]. In this study, a novel wheat–tetraploid *Th. elongatum* disomic substitution line (K17-1065-4) highly resistant to stripe rust at the adult stage was generated from a cross involving 8801, SM482, and SM921 and self-crossing to F_5_. We also developed and validated new specific PCR-based molecular markers on the basis of the whole-genome sequence of diploid *Th. elongatum* to efficiently trace the tetraploid *Th. elongatum* chromosome 6E during the breeding of disease-resistant wheat lines and identify tetraploid *Th. elongatum* chromosomes along with the chromosomes of other wheat-related species.

## 2. Results

### 2.1. Chromosomal Composition of K17-1065-4

**Figure 1:**
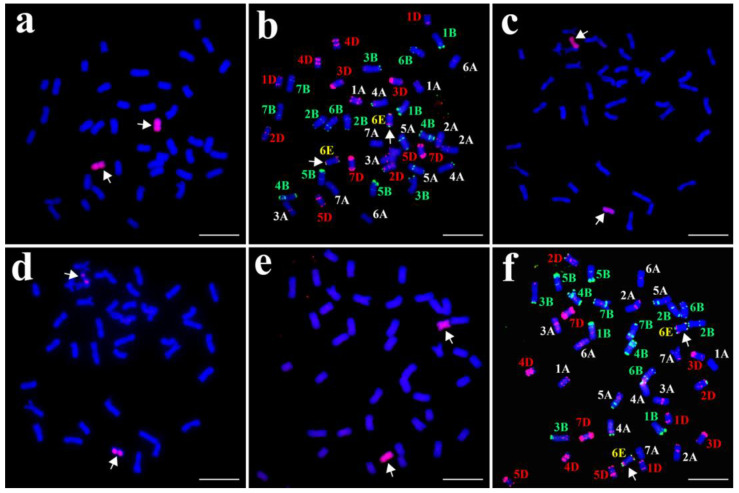
Identification of the wheat–tetraploid *Th. elongatum* substitution line K17-1065-4 using the GISH and FISH techniques. The probes were (**a**,**c**,**e**) tetraploid *Th. elongatum* genomic DNA; (**b**,**f**) Oligo-pSc119.2 (green) and Oligo-pTa535 (red); (**d**) Chr6 (red). Arrows indicate chromosomes 6E. Scale bar: 10 lm.

The GISH and FISH analyses were performed to clarify the chromosomal composition of the wheat–*Th. elongatum* line K17-1065-4. The GISH analysis involving tetraploid *Th. elongatum* genomic DNA as the probe and CS DNA as the blocker detected 40 wheat chromosomes and 2 E chromosomes in K17-1065-4 (Figure 1a). The FISH analysis of these 42 chromosomes was completed using Oligo-pSc119.2 (green) and Oligo-pTa535 (red), with the CS FISH karyotype and the tetraploid *Th. elongatum* E genome FISH karyotype serving as references [37,38]. The results suggested a pair of 6D chromosomes was replaced by a pair of 6E chromosomes in line K17-1065-4 (Figure 1b). Furthermore, a FISH chromosome painting analysis of the pair of E chromosomes in K17-1065-4 was performed using the E-genome-specific probes Chr1-Chr7. There was a strong signal for probe Chr6 (red) on the E chromosomes (Figure 1c,d). These results were consistent with the previously reported FISH karyotype of the tetraploid *Th. elongatum* 6E chromosomes [37]. Thus, K17-1065-4 is a wheat–tetraploid *Th. elongatum* 6E (6D) chromosomal substitution line.

To verify the cytological stability of K17-1065-4, 40 randomly selected seeds from the K17-1065-4 selfed progeny were characterized via GISH and FISH analyses. The results showed that all seeds carried two 6E chromosomes, but no 6D chromosomes (Figure 1e,f).

### 2.2. Response to Stripe Rust

**Figure 2:**
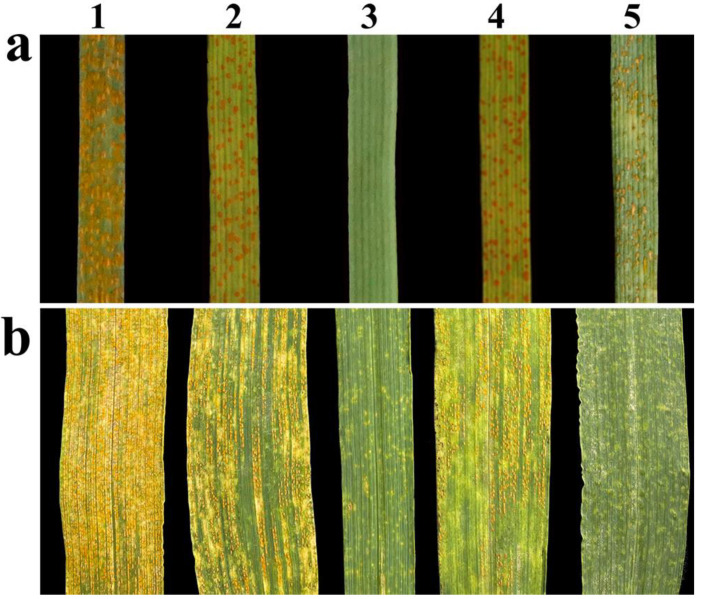
Stripe rust responses of wheat line K17-1065-4, its parents, and the control at (**a**) seedling stages and (**b**) adult plant stage. 1, SY95-71; 2, SM482; 3, 8801; 4, SM921; 5, K17-1065-4.

At the seedling stage, K17-1065-4 and its parents SM482 and SM921 were inoculated with *P. striiformis* f. sp. *tritici* race CYR-34 to evaluate stripe rust resistance. A wheat line SY95-71 was used as the susceptible control. The results indicated that SM482, SM921, and K17-1065-4 plants were susceptible to CYR-34 (IT = 8), whereas 8801 was immune to CYR-34 (IT = 0) (Figure 2a).

At the adult stage, SY95-71, SM482, SM921, 8801, and K17-1065-4 were inoculated with a mixture of *P. striiformis* f. sp. *tritici* races. Both SM482 and SM921 were susceptible to stripe rust (IT = 8), whereas 8801 and K17-1065-4 were highly resistant to stripe rust (IT = 1) (Figure 2b).

### 2.3. Agronomic Trait Evaluation

**Figure 3:**
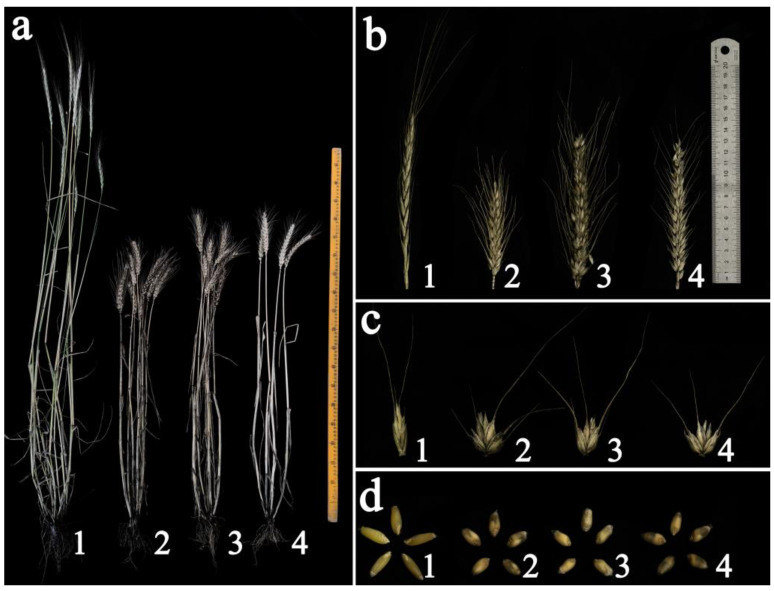
Plant morphology of wheat line K17-1065-4 and its parents. (**a**) Adult plants; (**b**) spikes; (**c**) spikelets; (**d**) grains. 1, 8801; 2, SM482; 3, SM921; 4, K17-1065-4.

K17-1065-4, SM482, SM921, and 8801 plants were assessed for agronomic traits in a research field at Sichuan Agricultural University. The tiller number, plant height, and spike length of K17-1065-4 were similar to those of SM482 and SM921, but were significantly lower than those of 8801 (Table 1; Figure 3). The grain number per spike of K17-1065-4 was significantly higher than that of all parents, and its 1000-grain weight was lower than that of SM921, not significantly different from that of SM482, and significantly higher than that of 8801.

### 2.4. Development and Validation of Specific Molecular Markers

A total of 83,685 SSR sequences were obtained by analyzing the 6E genomic sequence of diploid *Th. elongatum*. Primers were designed for all SSR sequences using Primer3 (https://primer3.ut.ee/) (accessed on 7 June 2022). The mock e-PCR amplification of the whole genomes of CS and diploid *Th. elongatum* indicated 20,738 pairs of primers could exclusively amplify the target fragments on the diploid *Th. elongatum* 6E chromosome. The SSR sequences corresponding to these markers were used to screen the other chromosomes (1E–5E and 7E) of diploid *Th. elongatum*, which revealed 8708 sequences with mismatches. Furthermore, these sequences were compared with the whole-genome sequence of CS; the sequences with ≥10% homology were discarded. Finally, 3382 unique SSR sequences were obtained, which were specific to the 6E chromosome of diploid *Th. elongatum*.

**Figure 4:**
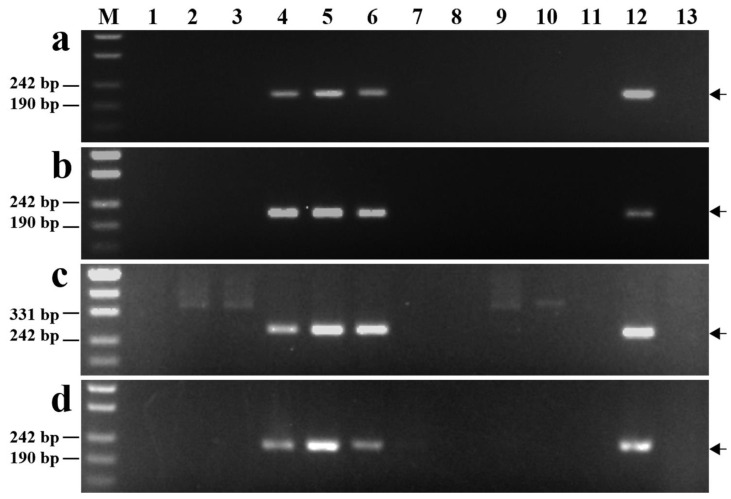
Amplification of markers. (**a**) *Chr6E-10*; (**b**) *Chr6E-24*; (**c**) *Chr6E-27*; (**d**) *Chr6E-48*; M, marker (500 bp); 1, CS; 2, SM482; 3, SM921; 4, *Th. elongatum*; 5, tetraploid *Th. elongatum*; 6, 8801; and 7 to 13, 1 to 7E substitution lines. Arrows show the diagnostic amplification products of tetraploid *Th. elongatum* 6E chromosome.

We randomly selected 60 specific SSR sequences located at different positions on chromosome 6E to be used as SSR markers (Table S2). The primer pairs for 33 SSR markers, including *Chr6E-10*, *Chr6E-24*, *Chr6E-27*, and *Chr6E-48*, amplified specific fragments in diploid *Th. elongatum*, tetraploid *Th. elongatum*, 8801, and K17-1065-4, but not in CS, SM482, SM921, and six wheat–tetraploid *Th. elongatum* substitution lines (1E–5E and 7E) (Figure 4a–d). Therefore, these SSR markers were considered to be specific to chromosome 6E of tetraploid *Th. elongatum*. The success rate for the PCR-based marker development was 53.3%.

**Figure 5:**
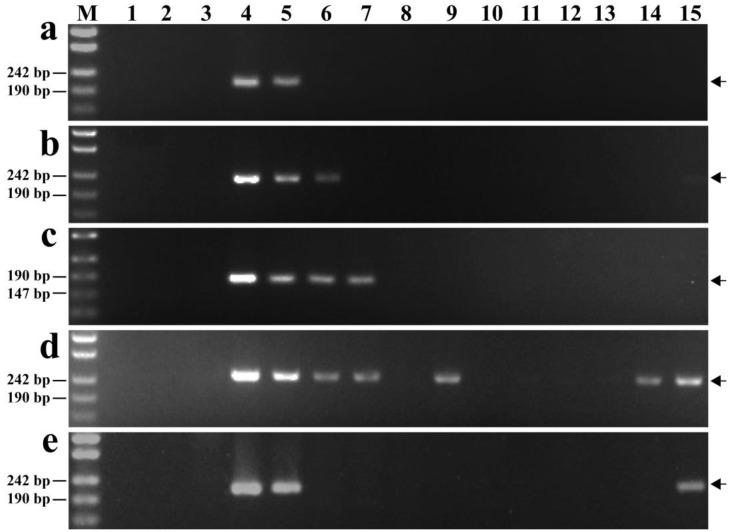
Stability and specificity markers. (**a**) *Chr6E-36*; (**b**) *Chr6E-48*; (**c**) *Chr6E-42*; (**d**) *Chr6E-2*; (**e**) *Chr6E-37*; M, marker (500 bp); 1, *T. monococcum*; 2, *Ae. speltoides*; 3, *Ae. tauschii*; 4, *Th. elongatum*; 5, tetraploid *Th. elongatum*; 6, *Th. ponticum*; 7, *Th. bessarabicum*; 8, *H. bogdanii*; 9, *Ag. cristatum*; 10, *S. cereale*; 11, *D. villosum*; 12, *Psathyrostachys huashanica*; 13, *Pseudoroegneria libanotica*; 14, *Th. caespitosum*; 15, *Psa. athericum*. Arrows show the diagnostic amplification products of tetraploid *Th. elongatum* 6E chromosome.

To evaluate the specificity and stability of these 33 markers, they were used for the PCR amplification involving 15 wheat relatives (Table S3). Ten amplified fragments were exclusive to diploid *Th. elongatum* and tetraploid *Th. elongatum* (Figure 5a). Additionally, the primer pairs for three markers amplified specific fragments only for diploid *Th. elongatum*, tetraploid *Th. elongatum*, and *Th. ponticum* (Figure 5b), while the primer pairs for another three markers amplified specific fragments only for diploid *Th. elongatum*, tetraploid *Th. elongatum*, *Th. ponticum*, and *Th. bessarabicum* (Figure 5c). The PCR analysis of other wheat relatives showed that 13, 2, 1, 2, and 7 markers amplified specific fragments from *Psa. athericum*, *Tri. caespitosum*, *Pse. libanotica*, *Ag. Cristatum,* and *Th. bessarabicum*, respectively (Figure 5d,e).

### 2.5. Utility of Specific Markers for Breeding

**Figure 6:**
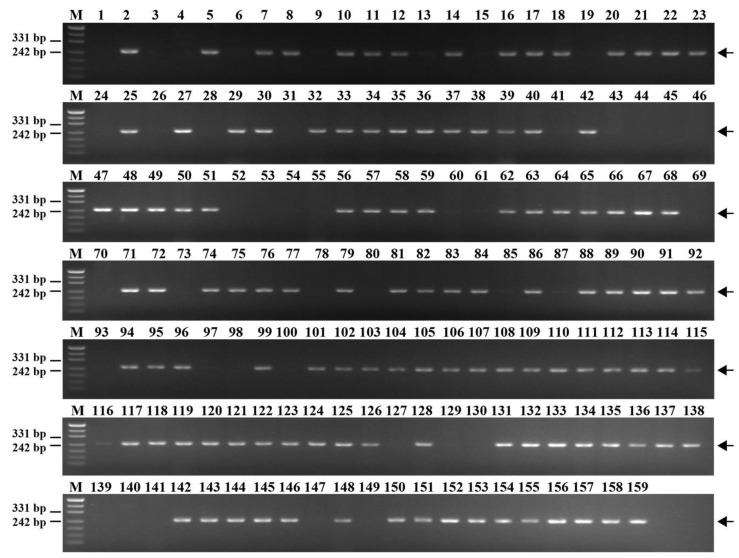
Presence and absence of marker *Chr6E-2* in 154 F_2_ plants of cross K17-1065-4/SM482. M, marker (500 bp); 1—CS; 2—8801; 3—SM482; 4—SM921; 5—K17-1065-4; 6—159, 154 F_2_ individuals. Arrows show the diagnostic amplification products of tetraploid *Th. elongatum* 6E chromosome.

**Figure 7:**
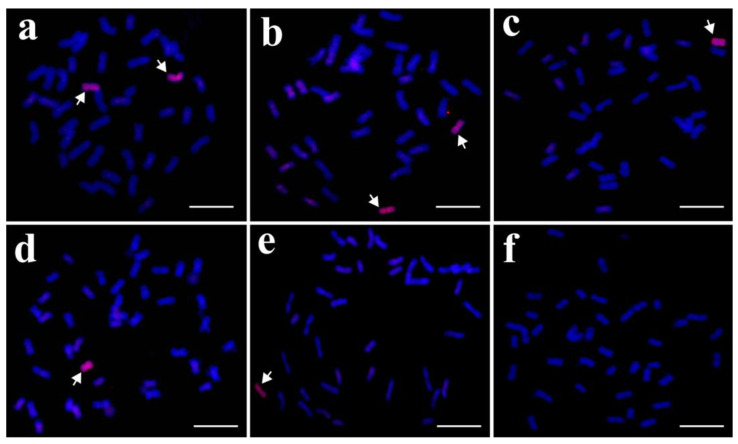
Identification of F_2_ plants from cross K17-1065-4/SM482 using genomic in situ hybridization. (**a**) F_2_-81; (**b**) F_2_-59; (**c**) F_2_-37; (**d**) F_2_-20; (**e**) F_2_-94; (**f**) F_2_-68. Arrows indicate chromosomes 6E.

**Figure 8:**
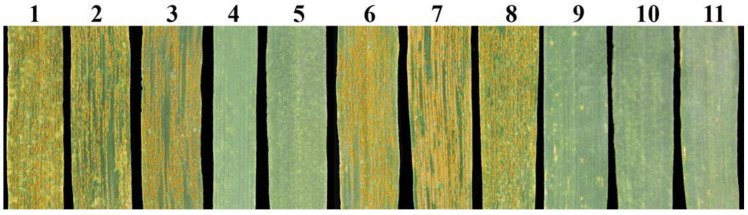
Assessment of K17-1065-4/SM482 F_2_ for stripe rust reactions at the adult stage. 1, SY95-71; 2, SM482; 3, SM921; 4, 8801; 5, K17-1065-4; 6 to 8, susceptible F_2_ plants; 9 to 11, resistant F_2_ plants.

To evaluate whether the 33 specific markers developed for the tetraploid *Th. elongatum* chromosome 6E were applicable for breeding, 154 F_2_ individuals obtained from the cross between K17-1065-4 and SM482 were selected for a molecular marker analysis, GISH analysis, and an assessment of stripe rust resistance. The PCR amplification results for the tetraploid *Th. elongatum* 6E-specific SSR markers revealed specific bands for 115 of the 154 plants (Figure 6). The GISH results indicated that these 115 plants contained one or two 6E chromosomes; the GISH signals were undetectable for the other 39 plants (Figure 7). Moreover, these 115 plants were highly resistant to stripe rust at the adult stage, whereas the other 39 plants were highly susceptible to stripe rust (Figure 8). Accordingly, these specific SSR markers may be useful for tracking stripe rust resistance genes linked to chromosome 6E of tetraploid *Th. elongatum*, making them potentially capable of wheat genetic improvement and breeding.

## 3. Discussion

*Th. elongatum* is a tertiary genetic resource that has many excellent agronomic traits useful for wheat crop improvement. Over the last several decades, several wheat–*Th. elongatum* lines have been produced via distant hybridizations. Ma et al. [39] assessed the stripe rust resistance of wheat–*Th. elongatum* substitution lines and mapped the dominantly inherited stripe rust resistance gene *YrE* to chromosome 3E. Jauhar [5] crossed diploid *Th. elongatum* with durum wheat Langdon to obtain one 1E addition line and two 1E substitution lines, which differed regarding Fusarium head blight infection rates. Another gene (*Yr6*9) mediating stripe rust resistance was identified in the wheat–*Th. ponticum* line CH7086 on the basis of stripe rust resistance and allele analyses [40]. Dai et al. [41] developed a wheat–rye–*Thinopyrum* tricentric hybrid by crossing 8801 with triticale T182, while also introducing the genes responsible for the resistance to Fusarium head blight, leaf rust, and stem rust into common wheat. In a recent study, two wheat–*Th. ponticum* substitution lines (ES-11 and ES-12) and a new translocation line were identified and characterized, and the stripe rust and stem rust resistance genes carried by *Th. ponticum* were successfully introduced into common wheat [42,43]. We previously reported that the wheat–tetraploid *Th. elongatum* 1E (1D) substitution line is highly resistant to stripe rust, the tetraploid *Th. elongatum* 3E chromosome carries salt tolerance genes, and the 4E (4D) disomic substitution line is resistant to stripe rust and powdery mildew at the seedling and adult stages [16,35,36]. Until now, there has been no report on the tetraploid *Th. elongatum* homologous group 6 heterochromosome lines. Therefore, we developed and characterized a novel wheat–tetraploid *Th. elongatum* 6E (6D) disomic substitution line K17-1065-4, which is highly resistant to stripe rust at the adult stage. In addition, the grain number per spike of K17-1065-4 was significantly higher than that of the parents, indicating that K17-1065-4 also carries genes associated with increased grain production. Therefore, K17-1065-4 represents a new excellent genetic resource for breeding disease-resistant wheat lines.

Several APR genes from the progenitors and wild relatives of wheat have been exploited, including *Yr36*, *Yr56*, and *Yr83* as well as a number of tentatively named genes and QTLs. Uauy et al. [44] first identified *Yr36* in *Triticum turgidum* ssp. *dicoccoides* plants exhibiting high-temperature adult plant resistance, with no detrimental effects on wheat yield. Subsequently, *Yr36* was effectively used by wheat breeders to produce wheat line Shumai 1701 [30]. Bansal and Bariana [45] identified the APR gene *Yr56* in durum wheat and determined it was located on chromosome 2AS bin 2AS5-0.87-1.00. Another APR gene (*Yr83*) was characterized by an in situ hybridization and molecular marker analysis of 10 6R chromosome deletion lines as well as 5 wheat-rye 6R chromosome translocation lines; the gene was mapped to the deletion bin of FL 0.73–1.00 of 6RL [24]. In addition, Zhang et al. [28] performed a bulked segregant RNA-seq analysis and mapped the APR gene *YrZ15-1370* from *Triticum boeoticum* to chromosome 6AL. More specifically, it was located within a 4.3 cM genetic interval flanked by KASP-1370-3 and KASP-1370-5, which corresponded to a 1.8 Mb physical region. The seeds of the *Ae. ventricosa* near-isogenic line AvS*Yr17*NIL were treated with EMS and the resulting F_2_ plants were analyzed; a novel recessive APR gene (*YrM1225*) was characterized and localized within a 7.5 cM interval on the short arm of chromosome 2A [27]. However, these genes or QTLs have not been adequately exploited by wheat breeders worldwide, and none of them are from *Th. elongatum*. In the present study, we developed the wheat–tetraploid *Th. elongatum* 6E (6D) substitution line K17-1065-4, which is highly resistant to multiple *Pst* races currently prevalent in China at the adult stage. A stripe rust resistance gene was localized on chromosome 6 of *Th. ponticum* [7], but the two genes were completely different in origin, genome, and response to *Pst* races, showing that they are two different stripe rust resistance genes. To the best of our knowledge, this is the first study to reveal the resistance to stripe rust mediated by chromosome group 6 of tetraploid *Th. elongatum*. Our results show that the stripe rust resistance of K17-1065-4 is conferred by a novel gene from tetraploid *Th. elongatum*. Moreover, this line may be a valuable resource for increasing the stripe rust resistance of wheat worldwide.

Molecular markers are important for the efficient detection of alien chromosomes or chromosomal fragments in wheat. Diverse and stable molecular markers provide an important foundation for breeding involving crosses between wheat varieties and wheat relatives. A number of molecular markers have been developed for *Th. elongatum* on the basis of RAPD, SSR, SCAR, AFLP, RGAP, GBS, and other techniques [35]. Two RAPD markers for CS and an ISSR marker for *Th. elongatum* were successfully transformed into SCAR markers for diploid *Th. elongatum*. Of these markers, two can specifically detect the E genome of *Th. elongatum*, whereas one is useful for tracking chromosomes 2E and 3E of *Th. elongatum* [46]. Chen et al. [1] developed 89 stable and specific molecular markers for *Th. elongatum* using SLAF-seq data. Additionally, many *Th. elongatum* SNP markers were obtained following a transcriptome sequencing analysis [47]. Furthermore, *Th. ponticum*-specific molecular markers were developed using SLAF-seq technology and used to construct a physical map of the 4Ag chromosome [48]. Li et al. [16] developed 132 markers for tetraploid *Th. elongatum* 1E by applying GBS technology. Another 74 markers were developed to accurately track stripe rust resistance genes on chromosome 4E of tetraploid *Th. elongatum* [35]. Although various *Th. elongatum*-specific molecular markers have been reported, most of these markers have not been precisely mapped to chromosomes or they are distributed in the terminal regions of chromosomes due to the previous lack of a *Th. elongatum* reference genome, which severely limits the localization and cloning of *Th. elongatum* genes associated with improved traits. In the current study, 33 SSR markers specific to tetraploid *Th. elongatum* chromosome 6E were developed according to the whole-genome sequence of diploid *Th. elongatum*. Validation of these specific markers in 15 wheat relatives showed that 10 of them accurately screened for the E genome carried by diploid and tetraploid *Th. elongatum* (Table S3). Only 11 pairs of markers amplified specific fragments in *Th. ponticum*, indicating that the genomes of *Th. ponticum* differed significantly from those of diploid and tetraploid *Th. elongatum* (Table S3), which is consistent with previous findings [16]. Only very few markers amplified specific fragments in the H, P, R, V, Ns, and St genomes, which indicated that the E genome of *Th. elongatum* and these genomes are genetically distant from one another. These specific markers are evenly distributed on chromosome 6E, making them potentially useful for identifying associated stripe rust resistance genes, and could also be employed by wheat breeding to help fine-map and clone the stripe rust resistance genes on chromosome 6E of tetraploid *Th. elongatum*.

## 4. Materials and Methods

### 4.1. Plant Materials

Line 8801 (2n = 6x = 42, AABBEE), which was derived from a cross between tetraploid *Th. elongatum* and *T. durum*, is highly resistant to wheat stripe rust, Fusarium head blight, and powdery mildew (kindly donated by Dr. George Fedak, Eastern Cereal and Oilseed Research Center, Ottawa, Canada). *T. durum* is highly susceptible to wheat stripe rust, which suggests that 8801’s stripe rust resistance is derived from the tetraploid *Th. elongatum* (Figure S1). The tetraploid *Th. elongatum* (2n = 4x = 28, EEEE, PI 531750) is a homozygous tetraploid formed by natural doubling [18,49,50]. The wheat cultivar Shumai 482 (SM482) and Shumai 921 (SM921) are susceptible to stripe rust. Wheat line SY95-71 was used as a stripe rust susceptible control. The wheat–tetraploid *Th. elongatum* disomic substitution line K17-1065-4 was obtained from the 8801/SM482//SM921 F_5_ progeny. The following seven wheat–tetraploid *Th. elongatum* disomic substitution lines derived from a cross between 8801 and a Sichuan wheat variety (some data not published) were used for developing molecular markers: 1E (1D), 2E (2A), 3E (3D), 4E (4D), 5E (5D), 6E (6D), and 7E (7D) substitution lines. An F_2_ population (154 individuals) from the cross between K17-1065-4 and SM482 was used to evaluate the utility of the molecular markers. Finally, the molecular markers were validated using the following 15 wheat relatives (Table S1). All the above plant materials were stored at the Triticeae Research Institute, Sichuan Agricultural University, China.

### 4.2. Genomic In Situ Hybridization (GISH) and Fluorescence In Situ Hybridization (FISH) Analyses

Seeds were germinated in an incubator at 22 °C. Samples were treated with N_2_O for 2 h when their roots reached 1–2 cm, after which they were immediately fixed in 90% acetic acid for 5 min before being digested with pectinase and cellulase [51]. Slides for the in situ hybridization were prepared as described by Han et al. [52]. Tetraploid *Th. elongatum* genomic DNA labeled with dUTP-ATTO-550 (Jena Bioscience, Jena, Germany) via nick translation was used as the probe and Chinese Spring (CS) DNA was used as the blocker (ratio of 1:150). The GISH analysis was performed according to a published method that was modified slightly [53]. Briefly, 1 µL tetraploid *Th. elongatum* genomic DNA probe, 3 µL CS DNA, and 16 µL hybridization mixture (1 g dextran sulfate, 5 mL formamide, 1 mL 20× SSC, 1 mL salmon sperm, and 2 mL ddH_2_O) were mixed and added dropwise to the slides. The samples were denatured at 85 °C for 5 min and then incubated overnight at 50 °C. They were subsequently washed with 2× SSC at 50 °C for 20 min and then with 75%, 95%, and 100% ethanol for 1 min each. The chromosome counterstaining and the slide microscopy were performed as described by Gong et al. [35].

After the GISH analyses, the samples were washed with 2× SSC for 30 min and then with 75% and 100% ethanol for 5 min each before being placed under bright light. The Oligo-pSc119.2 and Oligo-pTa535 FISH probes were added after the GISH signal was removed to determine the K17-1065-4 chromosomal composition. The FISH analyses were performed as described by Li et al. [16]. The FISH signals were recorded in the same way as the GISH signals.

### 4.3. FISH Chromosome Painting Analysis

The homologous group relationship of the exogenous chromosome carried by K17-1065-4 was determined by performing a FISH chromosome painting analysis using the bulk oligonucleotide libraries Chr1-Chr7 (provided by Prof. H.Q. Zhang, Triticeae Research Institute, Sichuan Agricultural University; data not published) for the whole-genome sequence of diploid *Th. elongatum*. The washed slides were used for the FISH analysis involving Oligo-pSc119.2 and Oli-gop-Ta535 to distinguish the chromosome composition of K17-1065-4. The FISH chromosome painting method was previously described by Bi et al. [54] and Han et al. [55]. The slides were washed as described by Komuro et al. [51]. Photomicrographs were taken as described in the FISH protocol.

### 4.4. Stripe Rust Resistance Evaluation

Seedling stripe rust reactions of K17-1065-4, 8801, SY95-71, SM482, and SM921 were evaluated under laboratory conditions at Sichuan Agricultural University, China. They were inoculated with *P. striiformis* f. sp. *tritici* race CYR-34 in an artificial climate chamber, Plants were inoculated at the two-leaf stage and the reaction to stripe rust was evaluated on the first leaf of each plant 15–18 days after inoculation [16]. SY95-71 was used as a susceptible control. and the disease resistance statistics were conducted as described by Line and Qayoum [56].

Adult-stage stripe rust resistance was examined in an experimental field at Sichuan Agricultural University, Chengdu, Sichuan, China. Specifically, a mixture comprising various *P. striiformis* f. sp. *tritici* races (CYR 32, CYR 33, CYR 34, Sull-4, Sull-5, Sull-7, and G22-14) was used to inoculate the fresh young leaves of SY95-71, K17-1065-4, 8801, SM482, and SM921 plants at the tillering stage according to the smear method, with talc added at a ratio of 1:50 (*Pst* races:talc, m/V) [57], the avirulence/virulence classification of the *Pst* races is provided in Table S4 [58]. Additionally, the infection types (ITs) of K17-1065-4 and its parents were assessed using the 0–9 scale described by Line and Qayoum [56], where plants with ITs of 0–1 were considered immune, ITs of 2–6 were moderately resistant, ITs of 7–8 were susceptible, and ITs of 9 were highly susceptible.

### 4.5. Agronomic Trait Evaluation

K17-1065-4, 8801, SM482, and SM921 plants were evaluated in terms of their agronomic traits during the 2020–2022 growing seasons in the experimental fields at Sichuan Agricultural University. The experiment was conducted using a randomized complete block design with three replications. Briefly, 15 seeds were sown in 2 m rows, with 0.3 m between rows. K17-1065-4 and its parents were assessed for agronomic traits including tiller number, spikelet number, spike length, plant height, grain number per spike, and 1000-grain weight. The data were analyzed using SPSS Statistics 24.0 software.

### 4.6. Development of Simple Sequence Repeat (SSR) Markers

The SSRs in the diploid *Th. elongatum* chromosome 6E sequence (NCBI BioProject ID PRJNA540081) were detected using the perl-based program MISA (http://pgrc.ipk-gatersleben.de/misa/download/misa.pl) (accessed on 7 June 2022) and the following criteria: single-nucleotide repeats of not less than 10; dinucleotide repeats of not less than 6; three to six nucleotide repeats of not less than 5; and two SSR loci separated by more than 100 bp. Primers were designed for all SSR sequences using Primer3 and then analyzed by performing an e-PCR mock amplification using the whole-genome sequences of CS and diploid *Th. elongatum*. Only the markers that amplified the target fragment on chromosome 6E of diploid *Th. elongatum* were selected. The SSR sequences corresponding to these markers were compared with the sequences of the other chromosomes (1E–5E and 7E) of diploid *Th. elongatum*. The sequences that were not complete matches were identified. These sequences were compared with the whole-genome sequence of CS and the sequences with ≥10% homology were removed to obtain unique SSR sequences specific to chromosome 6E of diploid *Th. elongatum*. All primers were produced by Sangon Biotech (Chengdu, China). The PCR amplification program and product detection were performed as described by Gong et al. [35].

### 4.7. Validation of Specific Molecular Markers

The specificity, repeatability, and stability of tetraploid *Th. elongatum* 6E chromosome-specific molecular markers were verified using 154 F_2_ individuals from the cross between K17-1065-4 and SM482 as well as 15 wheat-related species (Table S1). The PCR analysis was performed as described previously.

## 5. Conclusions

In the present study, we characterized a cytogenetically stable wheat–tetraploid *Th. elongatum* 6E (6D) disomic substitution line with a high level of resistance to stripe rust at the adult stage. It is an extremely valuable wheat germplasm resource for the development of new stripe-rust-resistant varieties. Moreover, 33 markers specific for tetraploid *Th. elongatum* chromosome 6E were developed based on the whole-genome sequence of diploid *Th. elongatum*. All these markers should be applicable for efficiently tracing tetraploid *Th. elongatum* chromosome 6E and its chromosomal segments during wheat-disease-resistant breeding. In the future, we will use ^60^Co-γ ionizing irradiation and CS*ph1b* mutants to induce this substitution line and produce small segmental translocations carrying stripe rust resistance genes for further breeding applications.

## Figures and Tables

**Table 1:** The agronomic traits of K17-1065-4 and its parental lines ^Z^.

Line	Growth Season	Tiller Number	Plant Height (cm)	Spike Length (cm)	Spikelet per Spike	Grains per Spike	1000-Grain Weight (g)
8801	2020–2021	8.8 ± 3.2 a	136.5 ± 5.4 a	16.4 ± 2.9 a	16.4 ± 3.0 b	17.0 ± 3.0 d	22.3 ± 0.8 c
	2021–2022	6.0 ± 0.7 a	121.5 ± 1.4 a	16.6 ± 1.1 a	20.6 ± 1.1 c	18.8 ± 1.3 c	20.6 ± 0.3 c
SM482	2020–2021	5.8 ± 1.1 b	79.9 ± 3.9 b	11.64 ± 0.7 b	22.4 ± 1.3 a	59.4 ± 4.3 c	27.5 ± 0.9 b
	2021–2022	5.0 ± 1.9 a	70.8 ± 2.7 d	13.8 ± 1.1 bc	23.0 ± 0.7 b	64.0 ± 3.2 b	27.4 ± 0.4 b
SM921	2020–2021	3.6 ± 0.9 b	82.0 ± 4.8 b	10.5 ± 0.9 b	21.4 ± 1.7 a	68.6 ± 6.6 b	34.1 ± 0.7 a
	2021–2022	6.0 ± 1.6 a	74.0 ± 2.5 c	15.1 ± 0.6 b	23.8 ± 1.1 ab	66.6 ± 3.6 b	33.6 ± 0.9 a
K17-1065-4	2020–2021	4.2 ± 1.3 b	79.1 ± 4.3 b	9.9 ± 1.6 b	21.2 ± 1.6 a	75.4 ± 3.4 a	26.9 ± 0.6 b
	2021–2022	5.6 ± 1.7 a	78.3 ± 1.8 b	13.7 ± 1.2 c	25.0 ± 1.0 a	75.6 ± 1.5 a	26.9 ± 0.1 b

^Z^ Data in the columns indicate means ± standard errors. Different lowercase letters following the means indicate significant differences at the *p* < 0.05 levels.

## Data Availability

The whole-genome sequence of diploid *Th. elongatum* reported in this study can be found in the National Center for Biotechnology Information, NCBI BioProject ID PRJNA540081. Data supporting the results of this study are in the manuscript or Supplementary Materials.

## Supplementary Materials

The following supporting information can be downloaded at: https://www.mdpi.com/article/10.3390/plants12122311/s1, Figure S1: Stripe rust responses of 8801 and the controls at adult plant. 1, SY95-71; 2, T. durum; 3, tetraploid Th. elongatum; 4, 8801; Table S1: Wheat relatives used in this study; Table S2: PCR amplification results of tetraploid Th. elongatum molecular markers; Table S3: Specific amplification of 6E chromosome markers in wheat-related species; Table S4: The avirulence(A) /virulence(V) formula of the *Pst* races used in the present study [58].
