# Supplementary material for: Development and Characterization of a Novel Wheat–Tetraploid Thinopyrum elongatum 6E (6D) Disomic Substitution Line with Stripe Rust Resistance at the Adult Stage
Source: Plants (Basel). 2023 Jun 14;12(12):2311. doi: 10.3390/plants12122311 (PMC10305598; doi:10.3390/plants12122311)
Supplement: Supplementary file 1 [file plants-12-02311-s001.zip › Figure S1.pdf]

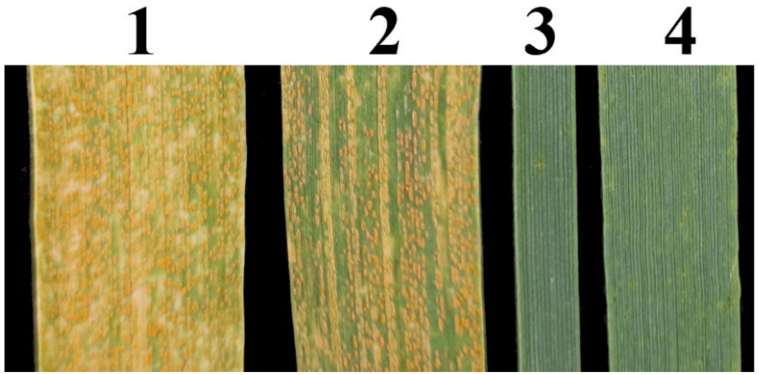

Figure S1. Stripe rust responses of 8801 and the controls at adult plant. 1, SY95-71; 2, *T. durum*; 3, tetraploid *Th. elongatum*; 4, 8801.
