# Supplementary material for: Development and Characterization of a Novel Wheat–Tetraploid Thinopyrum elongatum 6E (6D) Disomic Substitution Line with Stripe Rust Resistance at the Adult Stage
Source: Plants (Basel). 2023 Jun 14;12(12):2311. doi: 10.3390/plants12122311 (PMC10305598; doi:10.3390/plants12122311)
Supplement: Supplementary file 1 [file plants-12-02311-s001.zip › Table S1.pdf]

Table S1. Wheat relatives used in this study.

Species	Accession	Chromosome numbers	Genome
Triticum monococcum	PI427866	14	A^m
Triticum speltoides	PI542251	14	S
Triticum tauschii	AS84	14	D
Thinopyrum elongatum	PI531718	14	E
Thinopyrum elongatum	PI531750	28	E
Thinopyrum ponticum	PI531737	70	EEEStSt
Thinopyrum bessarabicum	W6-10232	14	E^bE^b
Pseudoroegneria libanotica	PI1228391	14	St
Dasypyrum villosum	PI251477	14	V
Hordeum bogdanii	Y1819	14	H
Agropyron cristatum	PI610892	14	P
Secale cereale	QL	14	R
Psathyrostachys huashanica	ZY3156	14	Ns
Trichopyrum caespitosum	PI634264	14	StE
Psammopyrum athericum	PI531744	42	StEP
