# Supplementary material for: Development and Characterization of a Novel Wheat–Tetraploid Thinopyrum elongatum 6E (6D) Disomic Substitution Line with Stripe Rust Resistance at the Adult Stage
Source: Plants (Basel). 2023 Jun 14;12(12):2311. doi: 10.3390/plants12122311 (PMC10305598; doi:10.3390/plants12122311)
Supplement: Supplementary file 1 [file plants-12-02311-s001.zip › Table S2.pdf]

Table S2. PCR amplification results of tetraploid *Th. elongatum* molecular markers.

Specific Markers	DNA Sequences of the Special Primers (5'-3')		Fragments Size（bp）	Annealing Temperatures (℃)	CS	SM482	SM921	8801	PI531718（E）	PI531750（EE）	1E（1D）	2E（2A）	3E（3D）	4E（4D）	5E（5D）	6E（6D）	7E（7D）
	Forward	Reverse															
chr6E-2	CAAATCAGCATCCAATCAGC	CGCTCGAGTTGCTAGTAGGC	267	61	0	0	0	1	1	1	0	0	0	0	0	1	0
chr6E-3	GGCAGAGTGGAAATCCAAAT	ATTGGTGCTACGGACCTCAC	242	58	0	0	0	1	1	1	0	0	0	0	0	1	0
chr6E-5	CTTGTGCTTGCCATGTGATT	TGACACATTGTTGTACGGGAA	196	58	0	0	0	1	1	1	0	0	0	0	0	1	0
chr6E-7	GTCGCCAGAGTCTCTTCGTT	CCTGGGAGAGCTCAGTTGAC	261	62	0	0	0	1	1	1	0	0	0	0	0	1	0
chr6E-8	AGTTCAAGTGCGGATAACGC	ATGCACTTGTGAGTGGAACG	197	61	0	0	0	1	1	1	0	0	0	0	0	1	0
chr6E-10	ACGAACCCTAAGAAAGGGGA	GGCATGTTGGACTGTAGCCT	217	60	0	0	0	1	1	1	0	0	0	0	0	1	0
chr6E-13	GCCCAGACATGGCCTAGTAA	CACGGCCCGTTTACTTTTT	163	62	0	0	0	1	1	1	0	0	0	0	0	1	0
chr6E-14	TTTTTCTCAGGCCCATGTGT	CTTTACCAAATCAGCCCCCT	241	58	0	0	0	1	1	1	0	0	0	0	0	1	0
chr6E-16	GCTCATCAATTTTTGAAGAAAAGG	CCGGTGAACCTTGTTGAAAT	191	56	0	0	0	1	1	1	0	0	0	0	0	1	0
chr6E-17	CCTTCCTCCCTTTCTCTGCT	CCACCACTCACATCTCTCCA	187	59	0	0	0	1	1	1	0	0	0	0	0	1	0
chr6E-21	GCCATAGGACAGACTGGAGC	CCCTCTCATCCCGTATCAAA	194	58	0	0	0	1	1	1	0	0	0	0	0	1	0
chr6E-23	ACCTGGGCCATGTTAGTCTG	TCAGTCGATGACCACTTTGC	109	59	0	0	0	1	1	1	0	0	0	0	0	1	0
chr6E-24	GAGCCCTCTTCTGTGCTGAC	ATCAACAGGTTTCCTGGACG	219	59	0	0	0	1	1	1	0	0	0	0	0	1	0
chr6E-25	TTAACCCCTGCAGAGCACTT	TTTGTATGCATGTAAACACCGA	138	57	0	0	0	1	1	1	0	0	0	0	0	1	0
chr6E-26	ACAGAACCGCGGGTTTAATA	AAAACACACCACGACTGCAA	143	57	0	0	0	1	1	1	0	0	0	0	0	1	0
chr6E-27	AAGTGGAGCGGCACATTTAC	CGTTGAACATGTTTCCAATACG	276	57	0	0	0	1	1	1	0	0	0	0	0	1	0
chr6E-28	TCACCTACTCGTCTTCGGCT	AGATGCTGGGCTCCTACTGA	280	61	0	0	0	1	1	1	0	0	0	0	0	1	0
chr6E-31	GCCGTTAGATCTGCTGTTCC	TAGAGAGAGGCCGTGCATTT	202	59	0	0	0	1	1	1	0	0	0	0	0	1	0
chr6E-36	TTCGCAAACAGAAATGGAGA	TGTGTGTCTTCGGTTATCGC	225	60	0	0	0	1	1	1	0	0	0	0	0	1	0
chr6E-37	ACCACATCCCGCGATATAAA	CTTGTCTCATCCTTCACGCA	214	57	0	0	0	1	1	1	0	0	0	0	0	1	0
chr6E-38	AGGAGGGGCATCACGTTAC	CCCTCCATGTCCTCATCATC	180	57	0	0	0	1	1	1	0	0	0	0	0	1	0
chr6E-40	GTCGCTTACCTTGTTCCCCT	ATATTTTTGGGCGAGTGGAG	142	57	0	0	0	1	1	1	0	0	0	0	0	1	0
chr6E-42	TATTGAGCAACCCCGTTTTC	TACTCCATCACGTCCCCTTC	187	57	0	0	0	1	1	1	0	0	0	0	0	1	0
chr6E-43	TCTGACCTTAAGCGCCAAGT	AAGGTACCCTACCGCCAACT	101	60	0	0	0	1	1	1	0	0	0	0	0	1	0
chr6E-45	TCTGTGCTGAAACTCGCACT	ACAAGTGAAACAACCCTGGC	252	59	0	0	0	1	1	1	0	0	0	0	0	1	0
chr6E-46	CCGAACTTGTTATCGTGGGT	GGCGCCAAATAAGGATTACC	193	57	0	0	0	1	1	1	0	0	0	0	0	1	0
chr6E-47	CTGGCTACGGCAGATGTGTA	AAATTACGGGATTGTCACGG	235	57	0	0	0	1	1	1	0	0	0	0	0	1	0
chr6E-48	GACACCAGCCTAACCCAAAA	CGCGCATTTAAAAATGGTTT	239	57	0	0	0	1	1	1	0	0	0	0	0	1	0
chr6E-49	ACGAGAGATTGGTGTCGGAG	GGGCGCAAATTTTAAAGACA	177	57	0	0	0	1	1	1	0	0	0	0	0	1	0
chr6E-50	ATACATGCCCACCAACCAAT	TCCCAACGGCTAGACTGACT	136	59	0	0	0	1	1	1	0	0	0	0	0	1	0
chr6E-51	CAGCGGTATTAATTGGCCTT	TGTGTCTTTGACGATTGGGA	175	58	0	0	0	1	1	1	0	0	0	0	0	1	0
chr6E-59	CATGCCCTGGCTAACTGTTT	CTTCACATCCCAAACACACG	136	59	0	0	0	1	1	1	0	0	0	0	0	1	0
chr6E-60	AGCCTGCGAGAGAAGAAGTG	CGCCGTTCAGTATCCTTTGT	122	59	0	0	0	1	1	1	0	0	0	0	0	1	0
