# Supplementary material for: Development and Characterization of a Novel Wheat–Tetraploid Thinopyrum elongatum 6E (6D) Disomic Substitution Line with Stripe Rust Resistance at the Adult Stage
Source: Plants (Basel). 2023 Jun 14;12(12):2311. doi: 10.3390/plants12122311 (PMC10305598; doi:10.3390/plants12122311)
Supplement: Supplementary file 1 [file plants-12-02311-s001.zip › Table S3.pdf]

Table S3. Specific amplification of 6E chromosome markers in wheat-related species.

Speicific Markers	PI427866 (A)	PI542251 (B)	AS84 (D)	PI531718 (E)	PI531750 (EE)	PI531737 (EEEStSt)	W6-10232 (E^b)	Y1819 (H)	PI610892 (P)	QL (R)	PI251477 (V)	ZY3156 (Ns)	PI228391 (St)	PI634264 (StE)	PI531744 (StEP)
chr6E-2	0	0	0	1	1	1	1	0	1	0	0	0	0	1	1
chr6E-3	0	0	0	1	1	1	0	0	0	0	0	0	0	1	0
chr6E-5	0	0	0	1	1	1	0	0	0	0	0	0	0	0	0
chr6E-7	0	0	0	1	1	1	1	0	0	0	0	0	0	0	0
chr6E-8	0	0	0	1	1	0	0	0	0	0	0	0	1	0	0
chr6E-10	0	0	0	1	1	1	0	0	0	0	0	0	0	0	1
chr6E-13	0	0	0	1	1	0	0	0	0	0	0	0	0	0	0
chr6E-14	0	0	0	1	1	0	0	0	0	0	0	0	0	0	0
chr6E-16	0	0	0	1	1	0	1	0	0	0	0	0	0	0	0
chr6E-17	0	0	0	1	1	0	0	0	0	0	0	0	0	0	0
chr6E-21	0	0	0	1	1	0	0	0	0	0	0	0	0	0	0
chr6E-23	0	0	0	1	1	0	0	0	0	0	0	0	0	0	1
chr6E-24	0	0	0	1	1	0	0	0	0	0	0	0	0	0	0
chr6E-25	0	0	0	1	1	1	0	0	0	0	0	0	0	0	0
chr6E-26	0	0	0	1	1	0	0	0	0	0	0	0	0	0	1
chr6E-27	0	0	0	1	1	0	0	0	1	0	0	0	0	0	0
chr6E-28	0	0	0	1	1	0	0	0	0	0	0	0	0	0	0
chr6E-31	0	0	0	1	1	0	0	0	0	0	0	0	0	0	1
chr6E-36	0	0	0	1	1	0	0	0	0	0	0	0	0	0	0
chr6E-37	0	0	0	1	1	0	0	0	0	0	0	0	0	0	1
chr6E-38	0	0	0	1	1	0	0	0	0	0	0	0	0	0	1
chr6E-40	0	0	0	1	1	0	1	0	0	0	0	0	0	0	1
chr6E-42	0	0	0	1	1	1	1	0	0	0	0	0	0	0	0
chr6E-43	0	0	0	1	1	0	0	0	0	0	0	0	0	0	1
chr6E-45	0	0	0	1	1	1	0	0	0	0	0	0	0	0	1
chr6E-46	0	0	0	1	1	0	0	0	0	0	0	0	0	0	0
chr6E-47	0	0	0	1	1	0	0	0	0	0	0	0	0	0	0
chr6E-48	0	0	0	1	1	1	0	0	0	0	0	0	0	0	0
chr6E-49	0	0	0	1	1	0	0	0	0	0	0	0	0	0	1
chr6E-50	0	0	0	1	1	1	1	0	0	0	0	0	0	0	0
chr6E-51	0	0	0	1	1	1	0	0	0	0	0	0	0	0	1
chr6E-59	0	0	0	1	1	0	0	0	0	0	0	0	0	0	0
chr6E-60	0	0	0	1	1	0	1	0	0	0	0	0	0	0	1
