# Supplementary material for: Development and Characterization of a Novel Wheat–Tetraploid Thinopyrum elongatum 6E (6D) Disomic Substitution Line with Stripe Rust Resistance at the Adult Stage
Source: Plants (Basel). 2023 Jun 14;12(12):2311. doi: 10.3390/plants12122311 (PMC10305598; doi:10.3390/plants12122311)
Supplement: Supplementary file 1 [file plants-12-02311-s001.zip › Table S4.pdf]

Table S4. The avirulence(A) /virulence(V) formula of the *Pst* races used in the present study [58].

Race name		Virulence or avirulence formula on *Yr* genes
CYR32	V:	*Yr1,Yr2,Yr3,Yr4,Yr6,Yr7,Yr8,Yr9,Yr11,Yr12,Yr13,Yr14,Yr16,Yr17,Yr18,Yr25,Yr27,Yr28,Yr29, Yr30,Yr31,Yr32,Yr43,Yr44,YrExp2,YrSp,Yr76,YrV23,YrA*
	A:	*Yr5,Yr10,Yr15,Yr19,Yr24,Yr26,Yr32,YrTr1,YrTye*
CYR33	V:	*Yr1,Yr2,Yr3,Yr4,Yr6,Yr7,Yr8,Yr9,Yr11,Yr12,Yr13,Yr14,Yr16,Yr17,Yr18,Yr25,Yr27,Yr28,Yr29, Yr30,Yr31,Yr32,Yr43,Yr44,YrExp2,YrA,YrSk,YrSp*
	A:	*Yr5,Yr10,Yr15,Yr19,Yr24,Yr26,Yr32,YrTr1*
CYR34	V:	*Yr1,Yr2,Yr3,Yr4,Yr6,Yr7,Yr8,Yr9,Yr10,Yr11,Yr12,Yr13,Yr14,Yr16,Yr17,Yr18,Yr19,Yr24,Yr25, Yr26,Yr27,Yr28,Yr29,Yr30,Yr31,Yr32,Yr43,Yr44,YrExp2,YrA,YrSk,YrSp,YrTye*
	A:	*Yr5,Yr15,Yr32,YrTr1*
G22–14	V:	*Yr1,Yr2,Yr3,Yr4,Yr6,Yr7,Yr8,Yr9,Yr10,Yr11,Yr12,Yr13,Yr14,Yr16,Yr19,Yr20,Yr24,Yr26*
	A:	*Yr3b,Yr4b,Yr5,Yr15,Yr17,Yr18*
Su11–4	V:	*Yr1,Yr2,Yr3,Yr4,Yr6,Yr7,Yr8,Yr9,Yr13,Yr14,Yr16,Yr18,Yr28,Yr29,Yr31,Yr43,Yr44,YrExp2*
	A:	*Yr5,Yr10,Yr11,Yr12,Yr15,Yr17,Yr19,Yr24,Yr26,Yr27,Yr32,YrSp,YrTr1*
Su11–5	V:	*Yr1,Yr2,Yr3,Yr4,Yr6,Yr7,Yr8,Yr9,Yr11,Yr14,Yr16,Yr18,Yr28,Yr29,Yr31,Yr43,Yr44,YrExp2,YrSp*
	A:	*Yr5,Yr10,Yr12,Yr13,Yr15,Yr17,Yr19,Yr24,Yr26,Yr27,Yr32,YrTr1*
Su11–7	V:	*Yr1,Yr2,Yr3,Yr4,Yr6,Yr7,Yr8,Yr9,Yr11,Yr12,Yr14,Yr16,Yr17,Yr18,Yr28,Yr29,Yr31,Yr43,Yr44,YrExp2,YrSp*
	A:	*Yr5,Yr10,Yr13,Yr15,Yr17,Yr19,Yr24,Yr26,Yr27,Yr32,YrTr1*
